# Improving maintenance efficiency and controlling costs in healthcare institutions through advanced analytical method

**DOI:** 10.1038/s41598-025-02176-8

**Published:** 2025-05-26

**Authors:** Kun Li, Lei Su, Jing Cheng, Yinyan Sun, Xinghua Ma

**Affiliations:** 3201 Hospital of Xi’an Jiaotong University Health Science Center, Hanzhong, Shaanxi 723000 China

**Keywords:** Medical equipment maintenance, Multi-parameter monitors, Expert experience, Fuzzy analytic hierarchy process, Fault tree analysis, Bayesian network, Maintenance costs, Health care economics, Biomedical engineering

## Abstract

This study aims to address the shortage of manpower and resources in the medical engineering departments of healthcare institutions while efficiently executing medical equipment maintenance and achieving controllable maintenance costs. In the absence of historical maintenance data, this research uses multi-parameter monitors as a case study. The methodology integrates Fault Tree Analysis (FTA) with the Fuzzy Analytic Hierarchy Process (FAHP) to combine expert judgments and address uncertainties in failure data. The results of this integration are then converted into a Bayesian Network (BN) for probabilistic reasoning and failure analysis. This comprehensive approach enables both qualitative and quantitative analysis of monitor failures across different usage stages (early, mid-term, and late). The analysis encompasses determining the failure probability at each stage, identifying high-risk components, examining the transition of failure modes, gaining insights into the aging characteristics of components, and developing preventive maintenance strategies. A cost-benefit analysis is conducted based on specific practical cases. This methodology successfully identified the failure probability of each component of the monitor at various stages, accurately pinpointed high-risk components, and provided a clear analysis of the transition of failure modes. Following one year of practical application, a significant reduction in costs was observed after implementing this method. The proposed approach effectively addresses the issue of low maintenance efficiency of medical equipment stemming from inadequate manpower and resources. It is particularly advantageous for healthcare institutions in developing countries and smaller medical facilities, significantly enhancing maintenance efficiency while controlling maintenance costs.

## Introduction

 The growing complexity of medical devices has significantly improved clinical outcomes^1,2^, however, it also presents substantial maintenance challenges, particularly in resource-limited settings^3,4^. Predictive and condition-based maintenance strategies have increasingly replaced traditional preventive approaches to minimize downtime and reduce costs^5^. Nevertheless, their implementation in developing countries faces systemic barriers^6^. For instance, 70% of donated medical equipment in Sub-Saharan Africa is non-functional due to a lack of trained personnel and unavailable spare parts^7^, while 50% of medical devices in low-income regions remain out of service^8^. These issues, compounded by fragmented manufacturer support and lengthy logistical delays^9^, highlight the urgent need for cost-effective and innovative solutions to balance maintenance quality and affordability.

In the field of medical equipment management, numerous studies have concentrated on the critical issue of medical equipment maintenance. Many prior investigations have conducted in-depth analyses of specific medical devices, yielding a series of valuable findings. For instance, Pinho, M. et al.^10^ proposed a multi-criteria ranking method for ventilators, thereby opening new avenues for their maintenance management. Li, J. et al.^11^utilized information fusion technology to deliver effective solutions for the maintenance and quality control of large medical equipment. Mellado-Silva, R. et al.^12^ designed a flow shop scheduling program aimed at addressing the challenge of arranging maintenance plans for medical equipment in public hospitals, with the primary goal of eliminating equipment downtime and minimizing total downtime. Hunte, J. L. et al.^13^employed a hybrid bayesian network to perform risk management on defibrillators. Lu, Q.P. et al.^14^ introduced the quality control circle to reduce both the downtime and frequency of linear accelerators. Verma, A. et al.^15^ conducted a comprehensive assessment of the failure probabilities of automatic blood analyzers throughout their life cycles. Additionally, beyond the application of specific technologies to analyze historical data, some researchers have also assessed medical equipment failures by drawing on expert experience. For example, 16 biomedical engineering technicians evaluated the failure modes and impacts of anesthesia machines^16^. Ayres-de‐Campos, D. et al.^17^collected information from various clinical and commercial sources to gain a comprehensive understanding of the availability of affordable basic obstetric equipment with low maintenance costs. They then conducted a systematic analysis of the maintenance costs associated with obstetric and gynecological medical equipment, providing a valuable reference for optimizing the allocation of maintenance resources. Taghipour, S. et al.^18^ precisely classified the failure types of infusion pumps and constructed an effective data analysis strategy at both the system and component levels, which aids in accurately identifying failure patterns of infusion pumps and implementing targeted maintenance measures. Despite the fruitful outcomes of the aforementioned studies, they do have certain limitations. On one hand, they did not fully address the issues of insufficient historical maintenance data and the excessive subjectivity inherent in expert judgments^19,20^. On the other hand, most studies tended to employ qualitative and quantitative analysis methods in isolation, failing to achieve comprehensive integration, which resulted in an incomplete understanding of the failure mechanisms^21^. Additionally, many studies overlooked the changes that occur at different stages of the medical equipment life cycle, where failure modes and probabilities may vary significantly^22^.

In light of these limitations, this study proposes a medical equipment maintenance strategy that effectively integrates qualitative and quantitative analysis methods, closely aligned with the actual operating conditions of medical institutions. This strategy aims to mitigate the subjectivity in expert judgments while considering the characteristics of different stages of the equipment life cycle, thereby enhancing the understanding of failure mechanisms and developing more effective maintenance strategies.

This study plans to adopt the analytic hierarchy process (AHP)^23^ and the fuzzy set method^24^, both of which have demonstrated effectiveness in various fields, to integrate diverse expert opinions and minimize uncertainties^25,26^. Specifically, by assigning weights to expert opinions based on their educational backgrounds, work experiences, and professional ranks, a more accurate aggregation of insights can be achieved. To address the subjectivity in expert judgment and the absence of sufficient historical data, this study combines fault tree analysis (FTA) and fuzzy AHP(FAHP), followed by a transformation into a Bayesian Network (BN)^27^. This integrated approach combines the system decomposition capabilities of FAHP-FTA^28–30^, the probabilistic reasoning advantages of BN, and the uncertainty management capabilities of fuzzy logic, thereby enabling effective handling of complex failure data and the uncertainties associated with expert judgments^31–33^.Furthermore, by examining different stages of the equipment life cycle (early, mid, and late stages), this study aims to formulate targeted maintenance strategies that optimize maintenance work and allocate resources effectively, ensuring the reliable operation of the equipment throughout its entire life cycle.

This paper presents a comprehensive analysis of strategies aimed at enhancing the maintenance of medical equipment. Utilizing the multi-parameter monitor at 3201 hospital as a case study, the research addresses practical aspects, including the integration of expert opinions through the application of the FAHP method. The study thoroughly considers expert perspectives from various dimensions, such as education and professional background, to improve the consistency and reliability of judgments. The integrated FAHP -FTA-BN method involves constructing a FTA model, which is then converted into a BN. This approach effectively tackles the challenges posed by uncertainty and imprecise data, thereby offering a more comprehensive and accurate pathway for analyzing failure probabilities. Additionally, the analysis based on life cycle stages entails a detailed investigation of the unique failure modes and probabilities associated with medical equipment at different stages of its life cycle, facilitating the development of more precise maintenance strategies. The formulation of these strategies is grounded in the analytical results derived from the life cycle stages, with the aim of enhancing efficiency and reducing costs while specifically addressing particular failure modes and resource constraints. Finally, empirical studies conducted in actual medical environments seek to validate the practicality and effectiveness of this method in minimizing maintenance costs and improving equipment reliability. By establishing a systematic analysis framework, this study aspires to provide feasible practical solutions for medical institutions facing limitations in equipment maintenance resources, thereby laying a solid foundation for the future advancement of medical equipment maintenance.

## Methods

**Fig. 1:**
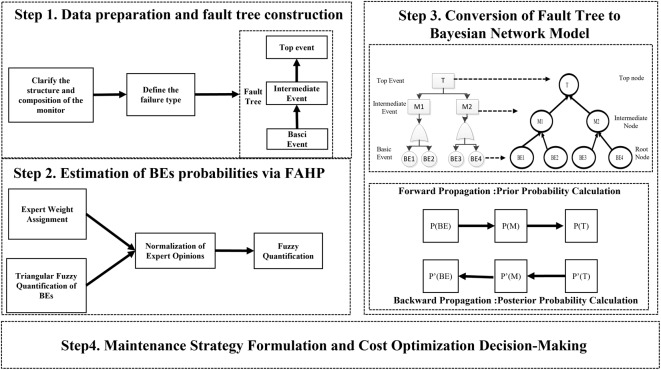
Flowchart of Research Steps.

To provide a clearer understanding of the research steps, a flowchart of the research steps, as shown in Fig. 1, is presented in this paper.

### Structure of multi-parameter monitors

Multi-parameter monitors are essential emergency medical devices widely used in hospitals. They continuously monitor vital signs in real time, including electrocardiograms (ECG), blood oxygen saturation, non-invasive blood pressure (NIBP), respiration, pulse, and body temperature. In addition to monitoring, they offer storage, display, analysis, and alarm functions. These devices significantly enhance the efficiency of medical staff and help maintain a high standard of medical care.

The main components of a multi-parameter monitor include signal acquisition, analog signal processing, digital signal processing, signal display, recording, and alarm systems. The signal acquisition components gather critical physiological signals via bioelectrodes and sensors, such as ECG, NIBP, and blood oxygen saturation, converting them into electrical signals. Analog signal processing components then refine these signals using circuits for impedance matching, filtering, and amplification, which effectively reduce noise and interference, thereby enhancing the signal-to-noise ratio.

The digital signal processing components primarily consist of an analog-to-digital converter, a microprocessor, and memory units. The analog-to-digital converter transforms analog signals into digital form. The memory stores operating programs, configuration data, and temporary data, while the microprocessor receives control information from the control panel and executes corresponding programs. Signal display, recording, and alarm components provide a user-friendly interface for information exchange between the device and medical staff. Medical professionals input monitoring parameters and requirements via the control panel, and the display presents monitored physiological parameters and waveforms. The recording section archives these parameters. When monitored parameters exceed preset ranges, the audio-visual alarm alerts medical staff to perform timely inspections or interventions.

### AHP-FTA-BN framework

#### Analytic hierarchy process

AHP is a structured decision-making method that decomposes complex problems into a hierarchical framework comprising objectives, criteria, and alternatives^23^. By performing pairwise comparisons at each level, AHP assigns relative weights to criteria and alternatives, ensuring consistency through eigenvector-based calculations. This approach is particularly effective in integrating expert judgments while minimizing subjectivity.

#### Fault tree analysis

FTA serves as a fundamental tool for assessing system reliability. It constructs a fault tree comprising events and logic gates, identifying the most critical system failure, referred to as the top event. This method traces back through events to uncover root causes, known as basic events^34^. Logic gates delineate the logical relationships between events, forming an inverted tree structure. Through quantitative calculations, analysts can determine the probability of occurrence for the top event. While the process of constructing a fault tree is straightforward, complex system architectures can result in intricate fault trees, complicating the calculations. Furthermore, FTA does not accommodate multi-state events.

#### Bayesian networks

BN offer a probabilistic graphical framework for modeling the relationships among random variables. Utilizing a directed acyclic graph, BN visually represent these relationships^35^. For example, a directed arc from X1 to X2 (X1 → X2) indicates a causal relationship, with X1 serving as the parent node and X2 as the child node. In this context, nodes represent variables, while directed edges illustrate dependencies, typically oriented from parent to child. Conditional probability tables articulate the logical interdependencies and facilitate the representation of multi-state events. BNs are adept at computing system reliability in a forward manner and assessing the influence of specific components in a backward direction. However, the construction of the network architecture and the process of data learning can often be complex.

#### Synergizing fault tree analysis and bayesian networks

FTA and BN each offer distinct advantages for reliability analysis. The integration of these methods can be especially advantageous for complex systems. Initially, a fault tree is constructed, which is subsequently translated into a BN for computational analysis. This integration circumvents the labor-intensive quantitative assessments typically associated with fault trees and streamlines the establishment of BN. Furthermore, BN is capable of accommodating various system states, and their bidirectional computation capabilities facilitate more comprehensive and precise reliability evaluations. Once the fault tree is developed, it can be transformed into a BN using specified mapping relationships, as illustrated in Table 1. In this transformation, events in the fault tree become nodes in the BN, with basic events represented as root nodes, intermediate events as intermediary nodes, and the top event as a leaf node. The logic gates are converted into conditional probability tables, while the occurrence probabilities of basic events in the FTA serve as the prior probabilities in the BN. Table 1 presents the mapping relationships between the fault tree and the BN.

**Table 1:** Mapping relation between fault tree and bayesian network.

Fault Tree		Bayesian Network	
Event	Top event	Node	Leaf node
	Intermediate event		Intermediate node
	Basic event		Root node
Logic gate		Conditional probability table	
Bottom event probability		Root node prior probability	

#### **fault identification and fault tree construction**

**Fig. 2:**
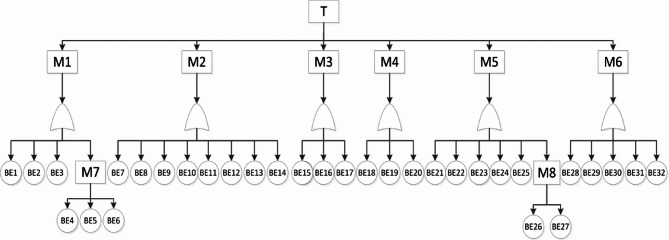
FTA model for Monitor.

Based on the structural principles of multi-parameter monitors, the top event (T) identified as “multi-parameter monitor not functioning properly” was determined through analysis of common failures. This analysis established eight common failure categories as secondary intermediate events (M1-M8). The failure causes are systematically analyzed layer by layer until all relevant basic events (BE1-BE32) are identified. Figure 2 illustrates the established fault tree, while Table 2 lists the corresponding fault codes and fault description.

**Table 2:** Expert judgments for each basic event.

Symbol	Events	Symbol	Events	Symbol	Events
BE1	Power switch key malfunction	BE15	ECG signal processing module failure	BE29	Oxygen Clip Electrode Failure
BE 2	Main control board failure	BE16	Faulty connection between the ECG signal acquisition lead wire and the ECG signal measurement module	BE30	Torn sphygmomanometer cuff
BE 3	Power switch to keypad connection failure	BE17	Faulty connection between the ECG signal measurement module and the main control board	BE31	Amplifier Failure
BE 4	Battery failure	BE18	SPO2 signal measurement module failure	BE32	Alarm system failure
BE 5	Faulty connection between the battery interface board and the power board	BE19	Faulty connection between the SPO2 signal acquisition extension cable and SPO2 signal measurement module	T	Multi-parameter monitor failure
BE 6	Power control failure	BE20	Faulty connection between the SPO2 signal measurement module and the main control board	M1	Power-on failure
BE7	Faulty connection between the keypad and the main control board	BE21	NIBP signal acquisition air leak in the airway	M2	Power supply module failure
BE8	Faulty connection between the power board to main control board failure	BE22	Air pump failure	M3	Display failure
BE9	Display malfunction	BE23	Faulty connection between the NIBP signal acquisition tube and NIBP signal measurementmodule	M4	EEG signal module failure
BE10	Backlight failure	BE24	NIBP signal acquisition guide tube bent and blocked	M5	SPO2 signal module failure
BE11	High-voltage board failure	BE25	Faulty connection between the NIBP signal measurement module and main control board	M6	NIBP signal module failure
BE12	Faulty connection between the Display screen and main control board	BE26	Solenoid valve blocked	M7	Solenoid valve failure
BE13	Faulty connection between the display screen and backlight board	BE27	Circuit board failure	M8	External sensor failure
BE14	Digital video interface failure.	BE28	ECG Electrode Failure		

#### Estimation of BEs probabilities

Historical data is typically utilized to quantify the probabilities of BE failure^36^. However, the predominance of qualitative case analyses over systematic and quantitative research in previous studies has resulted in a deficiency of failure data. Consequently, this study employs the expert judgment method. To address potential subjective differences arising from the experts’ varying educational backgrounds, work experiences, and professional ranks, the AHP is implemented to better integrate these perspectives. Weights are assigned to the ten participating experts based on their educational background (1–5 points), work experience (1–5 points), and professional rank (1–5 points). The weight table for the experts is presented in Table 2.

#### Triangular fuzzy quantification of BEs

**Table 3:** Expert classification and scores.

Attribute	Level	Score
Education Background	Ph.D.	5
	M.Sc.	4
	B.Sc.	3
	Junior college	2
	Secondary school and below	1
Experience(Exp.)	Exp.>30 years	5
	20 < Exp.≤30 years	4
	10 < Exp. ≤20 years	3
	5 < Exp.≤10 years	2
	Exp. ≤5 years	1
Professional Rank	Professorate senior engineer	5
	Senior Engineer	4
	Intermediate Engineer	3
	Assistant engineer	2
	Technician	1

Recognizing that fuzzy set theory effectively deals with uncertain and ambiguous information^24^, this study applies it to the quantification of BEs. The quantification process for BE probabilities includes four steps.

#### Triangular fuzzy quantification of BEs

**Fig. 3:**
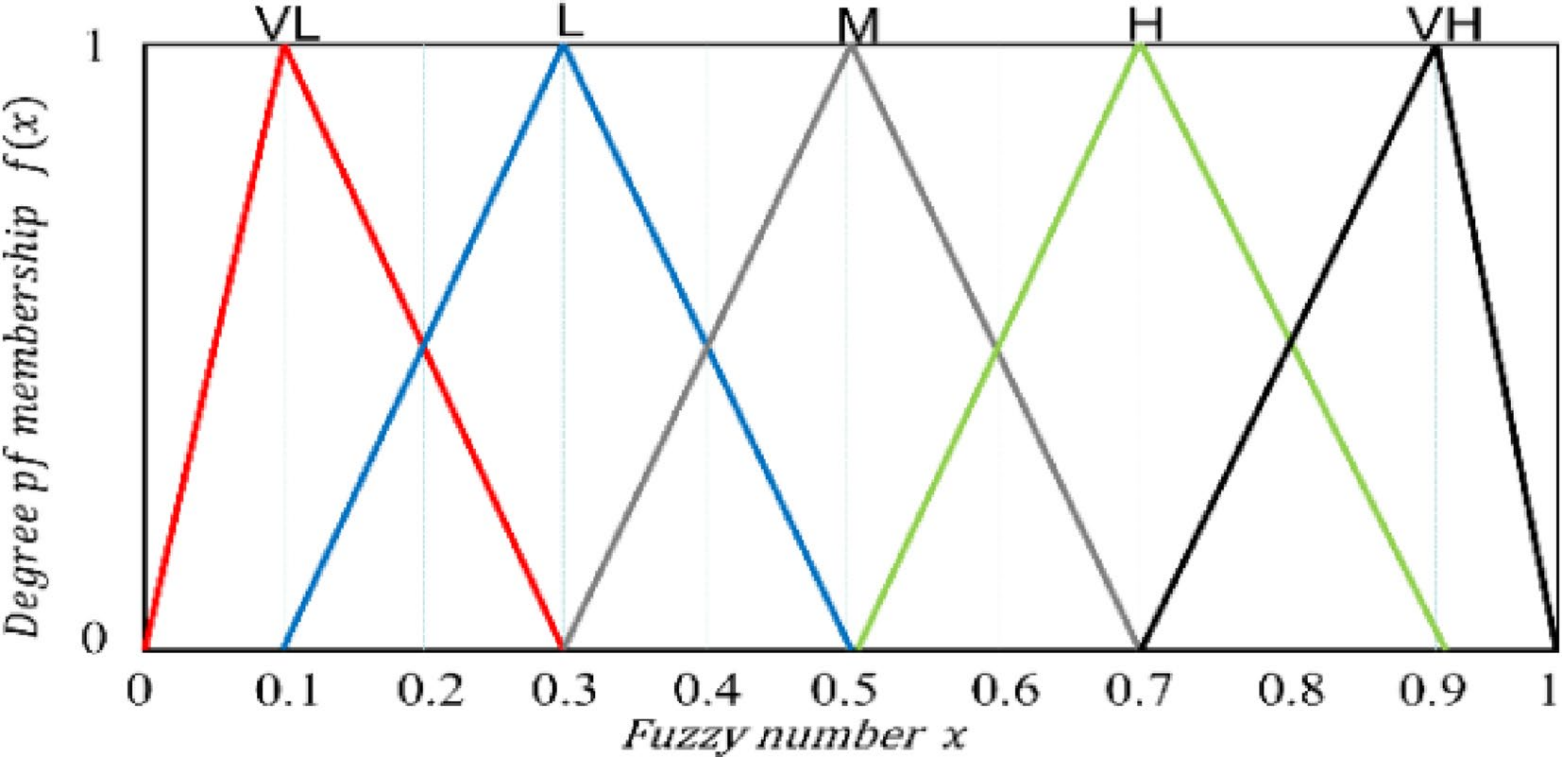
Corresponding numerical values for linguistic variables.

To streamline the modeling process, the study utilizes the common triangular membership function for natural language representation, enabling quantitative assessment. The linguistic variables used are Very Low (VL), Low (L), Medium (M), High (H), and Very High (VH), with values depicted in Fig. 3. The triangular fuzzy numbers (a_1_, a_2_, a_3_) and their corresponding membership function are shown in formula (1).

1 $$\:\begin{array}{c}\:\:\:\:\:\:\:f\left(x\right)=\left\{\begin{array}{c}\begin{array}{c}0;x\le\:{a}_{1}\\\:\left(x-{a}_{1}\right)/\left({a}_{2}-{a}_{1}\right)\\\:0;x\ge\:{a}_{2}\end{array};{a}_{1}\le\:x\le\:{a}_{2}\:\:\\\:\end{array}\right.\end{array}$$

#### Normalization of expert opinions

Experts’ varied backgrounds in monitor management may result in differing event failure judgments. To reconcile these differences, linguistic assessments expressed as fuzzy numbers across different experts must be combined into one unified fuzzy number. Traditional methods like those by Zhu^37^ depend on intersections within fuzzy sets of expert opinions. To overcome these constraints, this study introduces a new method for consistency aggregation, described in the steps that follow.

1. Calculate the degree of recognition between any two experts $\:{R}_{ij}$, which is computed using the formula below:

2 $$\:\begin{array}{c}{R}_{ij}=R\left({\theta\:}_{i},{\theta\:}_{j}\right)=1-\frac{1}{3}\sum\:_{k=1}^{3}\left|{a}_{ik}-{a}_{jk}\right|\end{array}$$

In this formula, $\:{\theta\:}_{i}=\left({a}_{i1,}{a}_{i2,}{a}_{i3,}\right)$ and $\:{\theta\:}_{j}=\left({a}_{j1,}{a}_{j2,}{a}_{j3,}\right)$ are the fuzzy numbers corresponding to experts $\:{E}_{i}$ and $\:{E}_{j}$, respectively. It is also easy to see that when $\:i=j$, $\:{R}_{ij}=1$.

(2) Calculate the recognition matrix M for all experts and the average recognition degree $\:A\left({E}_{i}\right)$ for each expert. The recognition matrix consists of elements $\:{R}_{ij}=R\left({\theta\:}_{i},{\theta\:}_{j}\right)$. The matrix M is shown as follows:

3 $$\:\begin{array}{c}M=\left[\begin{array}{cc}\begin{array}{ccc}{R}_{11}&\:{R}_{12}&\:\dots\:\\\:{R}_{21}&\:{R}_{22}&\:\cdots\:\\\:\vdots &\: \vdots &\:\ddots\:\end{array}&\:\begin{array}{c}{R}_{1n}\\\:{R}_{2n}\\\: \vdots \end{array}\\\:\begin{array}{ccc}{R}_{n1}&\:{R}_{n2}&\:\cdots\:\end{array}&\:{R}_{nn}\end{array}\right]\end{array}$$

The average recognition degree for each expert:

4 $$\:\begin{array}{c}A\left({E}_{i}\right)=\frac{1}{n-1}\sum\:_{j=1,j\ne\:i}^{n}{R}_{ij}\end{array}$$

(3) Calculate the Relative Agreement Degree (RAD) for each expert:

5 $$\:\begin{array}{c}{RAD}_{i}=A\left({E}_{i}\right)/\sum\:_{i=1}^{n}A\left({E}_{i}\right)\end{array}$$

In the formula above, n represents the number of experts, which is 10 in this study.

(4) Calculate the Importance Measure IM(E_i_) for each expert:

6 $$\:\begin{array}{c}IM\left({E}_{i}\right)=\frac{score\left(i\right)}{\sum\:_{i=1}^{n}score\left(i\right)}\end{array}$$

In this formula, score(i) is the importance score of E_i_, as referenced in Table 2.

(5) Calculate the Weight Coefficient W (E_i_​) for each expert:

The weight coefficient of an expert is a combined representation of IM and RAD, expressed by the following formula:

7 $$\:\begin{array}{c}w\left({E}_{i}\right)=\alpha\:IM\left({E}_{i}\right)+\left(1-\alpha\:\right){RAD}_{i}\end{array}$$

In this formula, α (0 ≤ α ≤ 1) is a relaxation factor that indicates the relative importance of importance measure compared to relative agreement degree. Based on the actual needs of this study, α = 0.5.

(6) Comprehensive result of expert judgments:

8 $$\:\begin{array}{c}{P}_{j}=\sum\:_{i=1}^{n}W\left({E}_{i}\right){P}_{ij},j=\text{1,2},\cdots\:,m\end{array}$$

In this formula, *P*_*j*_ is the comprehensive fuzzy result for basic event j, *P*_*ij*_ is the fuzzy number corresponding to expert *E*_*i*_, and *m* is the number of basic events.

#### Defuzzify the fuzzy possibility of BEs

The purpose of defuzzification is to transform expert opinions into fuzzy numbers, facilitating an efficient approximation of real-world systems. This study introduces the Left-Right fuzzy ranking method^38^, which is a widely adopted defuzzification technique in fuzzy mathematics. This method converts triangular fuzzy numbers into precise numerical values by considering both the left and right spreads, which represent pessimistic and optimistic uncertainties, respectively. Through a weighted aggregation of these spreads alongside the most probable value of the fuzzy number, the method calculates a defuzzified score that balances conservative and optimistic perspectives. For triangular fuzzy numbers, the calculation process can also be derived based on both the numerical characteristics and graphical representation of the fuzzy set. This detailed explanation not only provides clarity on the theoretical foundation of the Left-Right fuzzy ranking method but also highlights its practical computational framework, ensuring a comprehensive understanding of its implementation.

Convert fuzzy numbers into fuzzy possibility scores (FPS), expressed as follows:

9 $$\:\begin{array}{c}{FPS}_{L}=\frac{1-{a}_{1}}{1+{a}_{2}-{a}_{1}}\end{array}$$

10 $$\:\begin{array}{c}{FPS}_{R}=\frac{{a}_{3}}{1+{a}_{3}-{a}_{2}}\end{array}$$

*FPS* is derived from *FPS*_*L*​_ and *FPS*_*R*​_:

11 $$\:\begin{array}{c}FPS=\frac{{FPS}_{L}+1-{FPS}_{R}}{2}\end{array}$$

#### Convert FPS into fuzzy probability value of each BEs

The approximated efficiency score obtained after defuzzification can be converted to an approximate probability of failure using the following empirical formula:

12 $$\text{P}({\text{B}\text{E}}_{\text{i}})=\left\{\begin{array}{c}\begin{array}{c}\frac{1}{{10}^{{\left(1-\text{F}\text{P}\text{S}\right)}^{1/3}\times\:2.301}},FPS\ne\:0\\\:0,FPS=0\end{array}\:\\\:\end{array}\right.$$

In this formula, *P(BE*_*i*_*)* represents the approximate failure probability of *BE*_*i.*_

#### Conversion of fault tree to bayesian network model

A BN is an effective graphical model for representing and reasoning about uncertainties. By transforming a fault tree into a BN, we can efficiently calculate and analyze fault probabilities. In this transformation, each basic event in the fault tree corresponds to a node in the BN, as does the top event. Directed edges are established in the BN according to the logical gate relationships from the fault tree. When an AND gate connects multiple basic events to an intermediate or top event, directed edges for that event node in the BN will stem from all involved basic event nodes. For an OR gate, the directed edges will emanate from each basic event node linked to the OR gate. Conditional probability distributions for the BNs are then specified. Basic event nodes rely on prior probabilities for their distributions, while intermediate and top event nodes are calculated based on the fault tree’s logical gate relationships and basic event probabilities.

In this section, we provide a detailed explanation of the methodology for calculating the posterior probabilities of each BE given the failure of the monitor (P(T) = 1). This process involves two stages: (1) forward propagation to compute the prior probabilities of intermediate events (M) and the top event (T) and (2) backward propagation to determine the posterior probabilities of basic (BE) and intermediate (M) events conditioned on P(T = 1).

#### Forward propagation: prior probability calculation

(1) Basic Events (BE_i_):

The prior probabilities of the basic events (BE_i​_) are determined based on observational data or expert knowledge. These probabilities serve as the input to the Bayesian network and are assumed to be independent, i.e.:

13 $$\:\begin{array}{c}P\left({BE}_{i}\cap\:{BE}_{j}\right)=P\left({BE}_{i}\right)\cdot\:P\left({BE}_{j}\right)\:\:\:\:\:\:\forall\:\:i\ne\:j\end{array}$$

(2)Intermediate Events(M_j_):

Each intermediate event (M_j​_) depends probabilistically on a subset of basic events (BE_i​_). The prior probability of M_j​_ is computed using the conditional probability table (CPT) for M_j​_ and the joint probabilities of its related basic events. Through logical relationships such as OR gates, the conditional probability table of node M8 is established in Table 4, where P(M8​=1) and P(M8​=0) represent the occurrence and non-occurrence probabilities, respectively. Given the substantial number of intermediate nodes, the probability tables for other nodes are omitted.

14 $$\:\begin{array}{c}P\left({M}_{j}=1\right)=\sum\:_{b\in\:{\left\{\text{0,1}\right\}}^{r}}P\left({M}_{j}=1|BE=b\right)\cdot\:\prod\:_{k=1}^{r}P\left({BE}_{k}={b}_{k}\right)\end{array}$$

**Table 4:** Conditional probability table of node M8.

BE26	BE27	*P*(M8 = 1)	*P*(M8 = 0)
0	0	0	1
1	0	1	0
0	1	1	0
1	1	1	0

Where:

$\:P\left({M}_{j}=1|BE=b\right)$ is given in the CPT for $\:{M}_{j}$; *b = [b*_*1*_, *b*_*2*_,$\:\:\cdots\:$, *b*_*r*_*]* is the binary state vector of the *r* basic events affecting $\:{M}_{j}$; *P(BE*_*k*_ *= b*_*k*_*)* is know prior probability of *BE*_*k*_. This calculations performed for each intermediate event*(M*_*1*_, *M*_*2*_, $\:\cdots\:$, *M*_*q*_*)* in the network.

(3)Top Event(T):

The top event (T) represents the monitor failure and is probabilistically influence by all intermediate events (M_j_) in the network. The prior probability of T is computed marginalizing over all possible states of the intermediate events:

15 $$\:\begin{array}{c}P\left(T=1\right)=\sum\:_{m\in\:{\left\{0\:,1\right\}}^{q}}P\left(T=1|M=m\right)\cdot\:\prod\:_{j=1}^{q}P\left({M}_{j}={m}_{j}\right)\end{array}$$

Where: $\:P\left(T=1|M=m\right)$is the condition probability that *T = 1* given a special configuration of the intermediate events *m=[m*_*1*_, *m*_*2*_, $\:\cdots\:$, *m*_*q*_*]*. *P(M*_*j*_ *= m*_*j*_*)* is the prior probalility of *M*_*j*_ that was computed in formula (14).

Through this process, the probabilities of all nodes are propagated forward, allowing for the prior probabilities of intermediate and top events to be determined.

#### Backward propagation: posterior probability calculation

When the top event,*T*,is observed as *T = 1* (indicating monitor failure), Bayes’ theorem is applied to compute the posterior probabilities of the intermediate events(*M*_*j*_) and basic events(*BE*_*i*_). This represents backward inference within the Bayesian network.

1. Posterior probabilities of intermediate events (*M*_*j*_*)*.

The posterior probability of each intermediate event (*M*_*j*_)is calculate as:

16 $$\:\begin{array}{c}P\left({M}_{j}=1|T=1\right)=\frac{P\left(T=1|{M}_{j}=1\right)\cdot\:P\left({M}_{j}=1\right)}{P\left(T=1\right)}\end{array}$$

Where:

$\:P\left(T=1|{M}_{j}=1\right)$ is obtained from the conditional probality table of T. $\:P\left({M}_{j}=1\right)$ is the prior probability of *M*_*j*_ derived from formula (14). *P(T = 1)* is the prior probability of T derived from formula (15).

The posterior probability $\:P\left({M}_{j}=0|T=1\right)$ is calculated as :

17 $$\:\begin{array}{c}P\left({M}_{j}=0|T=1\right)=1-P\left({M}_{j}=1|T=1\right)\end{array}$$

(2) Posterior probabilities of Basic Events (*BE*_*i*_*)*.

The posterior probability of each basic events (*BEi*​) is computed based on its relationship with the intermediate events. Using the law of total probability, the posterior probability is given by:

18 $$\:\begin{array}{c}P\left({BE}_{i}=1|T=1\right)=\sum\:_{j}\left[P\left({BE}_{i}=1|{M}_{j}=1\right)\cdot\:P\left({M}_{j}=1|T=1\right)+P\left({BE}_{i}=1|{M}_{j}=0\right)\cdot\:P\left({M}_{j}=0|T=1\right)\right]\end{array}$$

Where:

$\:P\left({BE}_{i}=1|{M}_{j}=1\right)$ and $\:P\left({BE}_{i}=1|{M}_{j}=0\right)$ are know from CPT of$\:{\:M}_{j}$. $\:P\left({M}_{j}=1|T=1\right)$ is the posterior probability of $\:{\:M}_{j}$ calculated in formula (16). This calculation accounts for all intermediate events($\:{\:M}_{j}$) that influence a given basic events ($\:{BE}_{i}$).

#### Application of posterior probabilities

After calculating the posterior probabilities of basic events ($\:P\left({BE}_{i}=1|T=1\right)$), these probabilities are employed to identify and rank the failure-inducing factors across different lifecycle stages of the monitor. This ranking enables a systematic, dynamic determination of the dominant causes of failures at each stage, without relying on fixed thresholds to classify events as “high” or “low” impact. By focusing on the highest-ranked basic events within each stage, key failure mechanisms can be identified, guiding targeted interventions such as preventative maintenance, operational adjustments, or design optimizations tailored to specific lifecycle phases.

## Implementation

### Calculate the prior probabilities of BEs

As detailed in Sect. 2.3, information regarding monitor failures was obtained through consultations with 10 experts. These experts included engineers from the hospital’s medical equipment management department and product after-sales service teams. Their assessments varied due to differences in education, experience, and professional rank. To address these subjective variations, a weighting scheme, as presented in Table 2, was employed to better integrate the expert judgments. The experts evaluated monitors based on their years of use, categorized as follows: group A (1–5 years), group B (6–10 years), and group C (11–15 years). This method ensures a comprehensive assessment covering all hospital monitors. Table 5 details the expert profiles along with their respective weighting scores.

**Table 5:** The scores of expert importance.

Expert No.	Education Background	Experience	Professional Rank	score
1	4	3	5	12
2	4	3	3	10
3	4	3	3	10
4	3	5	5	13
5	3	5	4	12
6	3	4	3	10
7	3	3	3	9
8	3	3	3	9
9	3	2	3	8
10	2	4	1	7

This study uses group B’s BE1 to illustrate the expert opinion normalization process in detail. Drawing from expert evaluations of BE1 failure alongside linguistic variable quantification, the insights from 10 experts are translated into the following fuzzy numbers: (0, 0.1, 0.3), (0.3, 0.5, 0.7), (0, 0.1, 0.3), (0.1, 0.3, 0.5), (0.7, 0.9, 1.0), (0, 0.1, 0.3), (0, 0.1, 0.3), (0, 0.1, 0.3), (0.3, 0.5, 0.7), and (0, 0.1, 0.3).

$$\:M=\left[\begin{array}{cccccccccc}1&\:0.63&\:1&\:1&\:0.27&\:1&\:1&\:1&\:0.63&\:1\\\:0.63&\:1&\:0.63&\:0.63&\:0.63&\:0.63&\:0.63&\:0.63&\:1&\:0.63\\\:1&\:0.63&\:1&\:1&\:0.27&\:1&\:1&\:1&\:0.63&\:1\\\:1&\:0.63&\:1&\:1&\:0.27&\:1&\:1&\:1&\:0.63&\:1\\\:0.27&\:0.63&\:0.27&\:0.27&\:1&\:0.27&\:0.27&\:0.27&\:0.63&\:0.27\\\:1&\:0.63&\:1&\:1&\:0.27&\:1&\:1&\:1&\:0.63&\:1\\\:1&\:0.63&\:1&\:1&\:0.27&\:1&\:1&\:1&\:0.63&\:1\\\:1&\:0.63&\:1&\:1&\:0.27&\:1&\:1&\:1&\:0.63&\:1\\\:0.63&\:1&\:0.63&\:0.63&\:0.63&\:0.63&\:0.63&\:0.63&\:1&\:0.63\\\:1&\:0.63&\:1&\:1&\:0.27&\:1&\:1&\:1&\:0.63&\:1\end{array}\right]$$

The relaxation factor, which balances the importance of experts against the relative agreement degree, is obtained from formulas (4) to (7). This facilitates the computation of each expert’s average recognition degree, relative agreement degree, importance, and weight coefficient, as outlined in Table 5.

Using formula (8), the normalized expert judgment results for all component failures are illustrated in Table 6. The Left-Right fuzzy ranking method is employed for defuzzification, enabling the calculation of defuzzification scores and failure rates for each component in group B, based on formulas (11) and (12). These failure rates are used as prior probabilities in subsequent research, as listed in Table 7.

**Table 6:** Average recognition, relative recognition, importance, and weight coefficient of each expert.

Expert No.	A(Ei)	RADi	IM(Ei)	W(Ei)
1	0.84	0.11	0.12	0.115
2	0.67	0.09	0.12	0.105
3	0.84	0.11	0.09	0.10
4	0.84	0.11	0.13	0.12
5	0.35	0.05	0.12	0.085
6	0.84	0.11	0.1	0.105
7	0.84	0.11	0.9	0.10
8	0.84	0.11	0.9	0.10
9	0.67	0.09	0.7	0.08
10	0.84	0.11	0.7	0.09

**Table 7:** Calculation results of all BEs.

Basic Event	Aggregated fuzzy number M(a, b and c)			FPS	*P*(BEi)	Rank
BE1	0.1119	0.2381	0.4298	0.3899	0.002131	19
BE2	0.1092	0.2687	0.4687	0.4233	0.002812	14
BE3	0.07994	0.2415	0.4415	0.3815	0.001981	20
BE4	0.08902	0.2606	0.4606	0.4051	0.002424	16
BE5	0.06614	0.2151	0.4151	0.3482	0.001459	27
BE6	0.07844	0.2405	0.4405	0.3798	0.001951	21
BE7	0.1197	0.2792	0.4722	0.4356	0.0031	10
BE8	0.06814	0.2181	0.4181	0.3522	0.001516	26
BE9	0.1132	0.2722	0.4662	0.4258	0.00287	13
BE10	0.07164	0.2231	0.4231	0.359	0.001615	25
BE11	0.06514	0.2151	0.4151	0.3477	0.001452	28
BE12	0.04601	0.1754	0.3754	0.2984	0.0008718	32
BE13	0.1197	0.2782	0.4712	0.4346	0.003076	11
BE14	0.0505	0.201	0.401	0.3263	0.001174	31
BE15	0.1077	0.2667	0.4667	0.4205	0.002751	15
BE16	0.1192	0.2772	0.4697	0.4331	0.00304	12
BE17	0.051	0.202	0.402	0.3275	0.001189	30
BE18	0.112	0.2831	0.4762	0.4357	0.003103	9
BE19	0.07744	0.2385	0.4385	0.3773	0.001908	22
BE20	0.08529	0.2337	0.4276	0.3733	0.001841	24
BE21	0.052	0.204	0.404	0.33	0.00122	29
BE22	0.2051	0.3966	0.5966	0.5991	0.009716	4
BE23	0.2365	0.4081	0.6081	0.6263	0.01156	3
BE24	0.287	0.4782	0.6692	0.7173	0.02055	2
BE25	0.128	0.2995	0.4995	0.4635	0.003836	7
BE26	0.09271	0.2516	0.4516	0.398	0.002283	18
BE27	0.09421	0.2536	0.4536	0.4007	0.002337	17
BE28	0.1243	0.2954	0.4954	0.4576	0.003671	8
BE29	0.1841	0.3546	0.5546	0.5467	0.00689	5
BE30	0.3417	0.5237	0.6939	0.7796	0.03089	1
BE31	0.08319	0.2341	0.4341	0.3757	0.001882	23
BE32	0.1351	0.3065	0.4989	0.4703	0.004034	6

### Construction of the bayesian network for the multi-parameter monitor

**Fig. 4:**
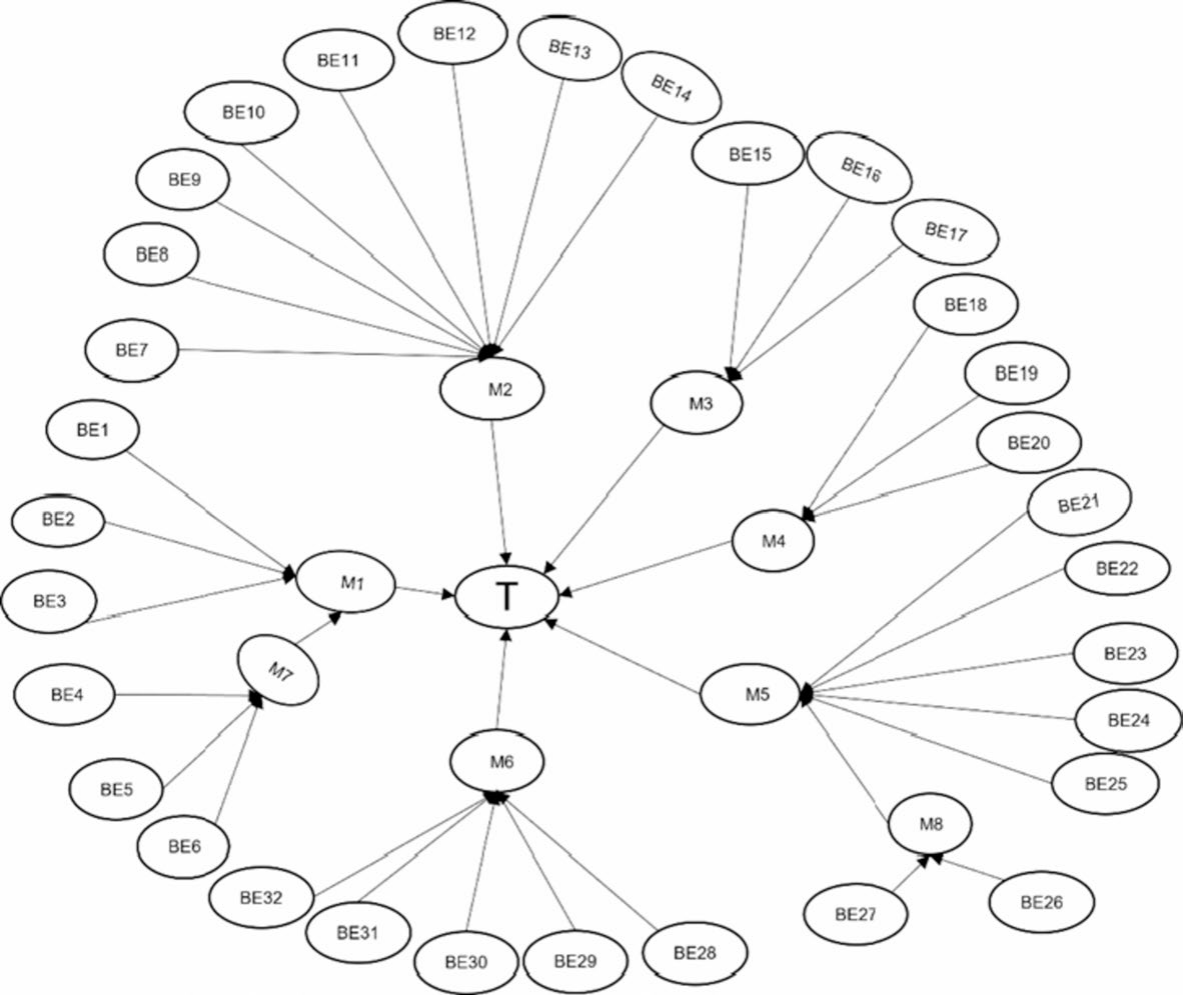
Bayesian network of the multi-parameter monitor.

**Fig. 5:**
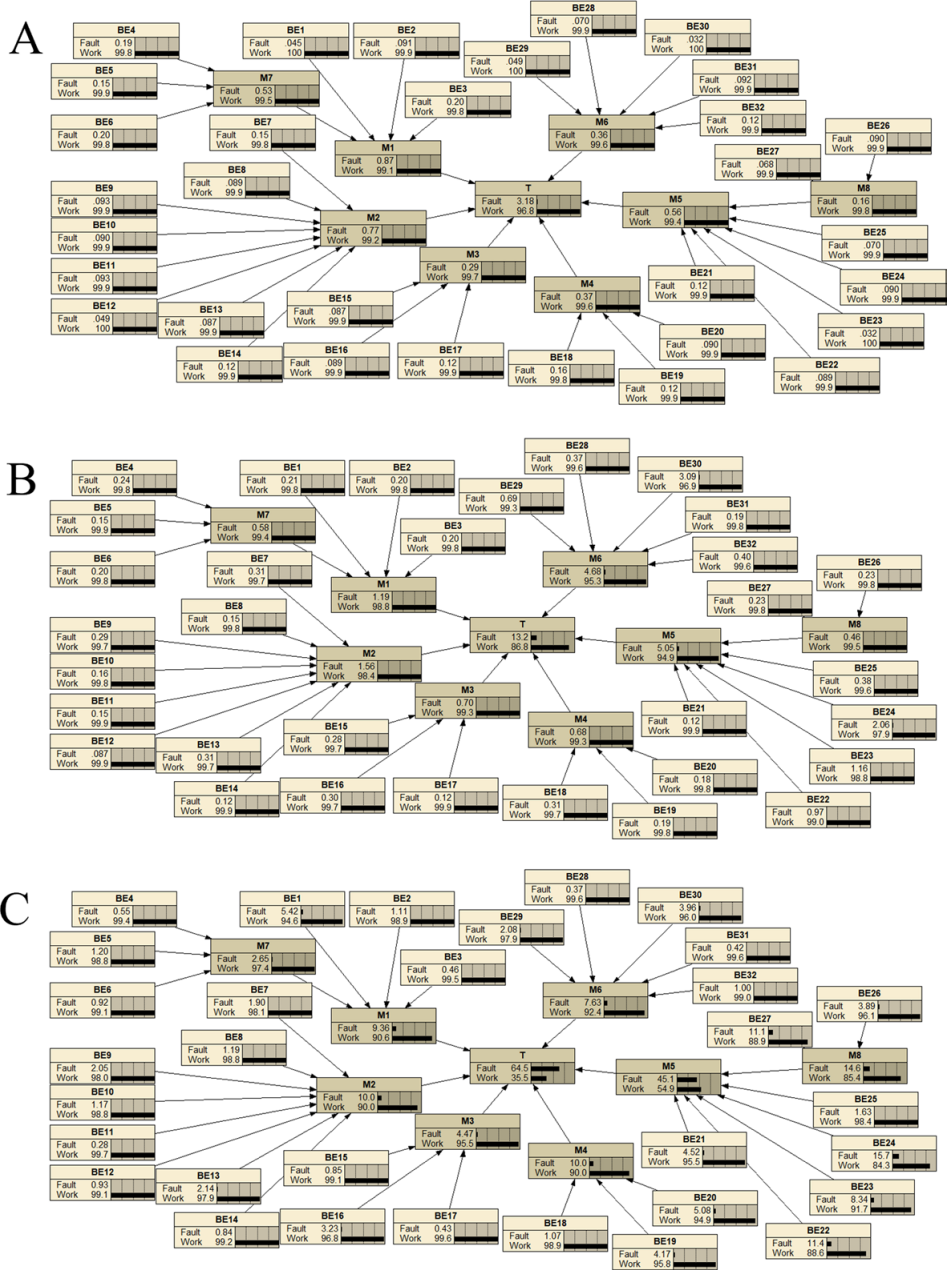
Calculation results of leaf node occurrence probabilities for multi-parameter monitors at different usage stages. This figure shows the prior probabilities of basic events (BE), intermediate events (M), and the top event (T) for multi-parameter monitors at three usage stages, calculated using the method in Sect. 2.4.1 (Forward Propagation: Prior Probability Calculation). Probabilities were derived from expert judgments based on original data collected in this study, calculated and visualized using the FAHP-FTA-BN framework and Netica (Version 7.01, Norsys Software Corp.). No external data or database was used. Panel A: Monitors (1–5 years). Panel B: Monitors (6–10 years). Panel C: Monitors (11–15 years). “Fault” indicates failure probability, and “Work” indicates normal operation probability. These results highlight failure trends across life-cycle stages, supporting targeted maintenance planning.

The fault tree of the multi-parameter monitor was converted into a BN, as shown in Fig. 4. Using the previously derived prior probabilities from the research data, failure probabilities for the monitor were calculated and visualized with Netica (Version 7.01, Norsys Software Corp.), as illustrated in Fig. 5. The failure probability for monitors used 1–5 years is 3.18%, for those used 6–10 years it is 13.2%, and for those used 11–15 years it rises to 64.5%. These results align logically with expectations, as failure rates increase over time. Particularly, once monitors surpass the manufacturer-recommended lifespan (e.g., beyond 10 years), the probability of failure escalates significantly.

Figure 5; Table 8 illustrate the tendencies of failure probabilities for different monitor components over various usage periods. In group A (1–5 years), components M1 and M2 are noteworthy; in group B (6–10 years), M5 and M6 stand out; and in group C (11–15 years), M2, M4, and M5 are prominent. By analyzing the failure rate trends across three operational periods, M3 (0.29%, 0.7%, 4.47%) and M7 (0.53%, 0.58%, 2.65%) demonstrate consistently low failure rates and minimal variability. This significant performance advantage compared to other components strongly validates their exceptional stability and reliability throughout the product lifecycle.

**Table 8:** Component failure rates (%) across monitor lifecycle stages.

Component	Groups A(%)	Groups B(%)	Groups C(%)	Mean ± SD(%)	CV (Coefficient of Variation)
M1	0.87	1.19	9.36	3.81 ± 4.81	1.26
M2	0.77	1.56	10	4.11 ± 5.12	1.24
M3*	0.29	0.7	4.47	1.82 ± 2.3	1.15
M4	0.37	0.68	10	3.68 ± 5.47	1.49
M5	0.56	5.05	45.1	16.9 ± 24.52	1.45
M6	0.36	4.68	7.63	4.22 ± 3.66	0.87
M7*	0.53	0.58	2.65	1.25 ± 1.21	0.97

**Fig. 6:**
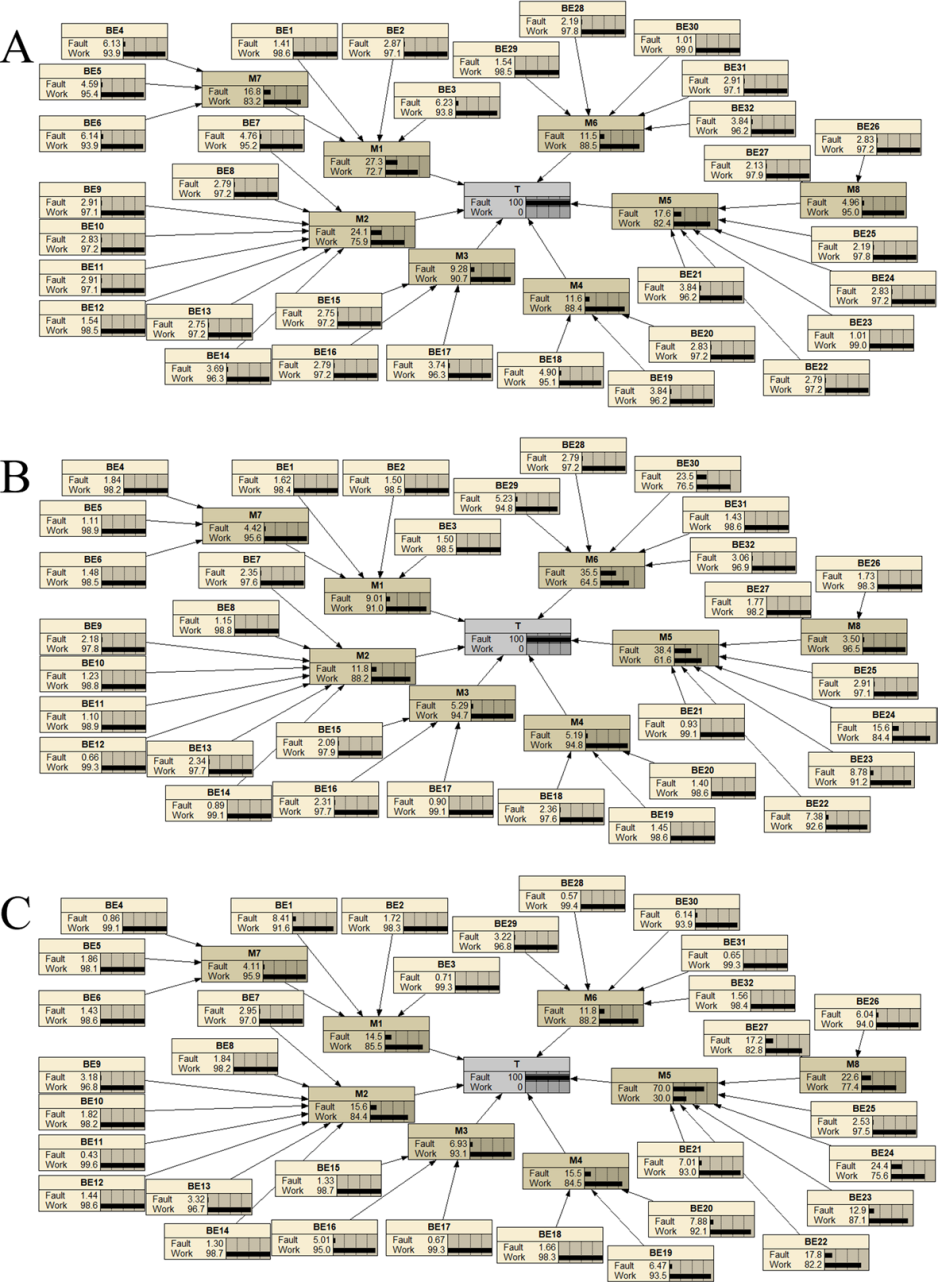
Posterior probabilities of events for the root node of multi-parameter monitors at different usage stages. This figure illustrates the posterior probabilities of basic events (BE), intermediate events (M), and the top event (T) for multi-parameter monitors under the failure condition (P(T = 1)) across three usage stages, calculated using the method in Sect. 2.4.2 (Backward Propagation: Posterior Probability Calculation). Probabilities were derived from expert judgments based on original data collected in this study, calculated and visualized using the FAHP-FTA-BN framework and Netica (Version 7.01, Norsys Software Corp.). No external data or database was used. Panel A: Monitors (1–5 years).Panel B: Monitors (6–10 years).Panel C: Monitors (11–15 years).“Fault” indicates failure probability, and “Work” indicates normal operation probability. These results reveal how failure conditions propagate through the system at different life-cycle stages, providing insights for system reliability assessment.

**Fig. 7:**
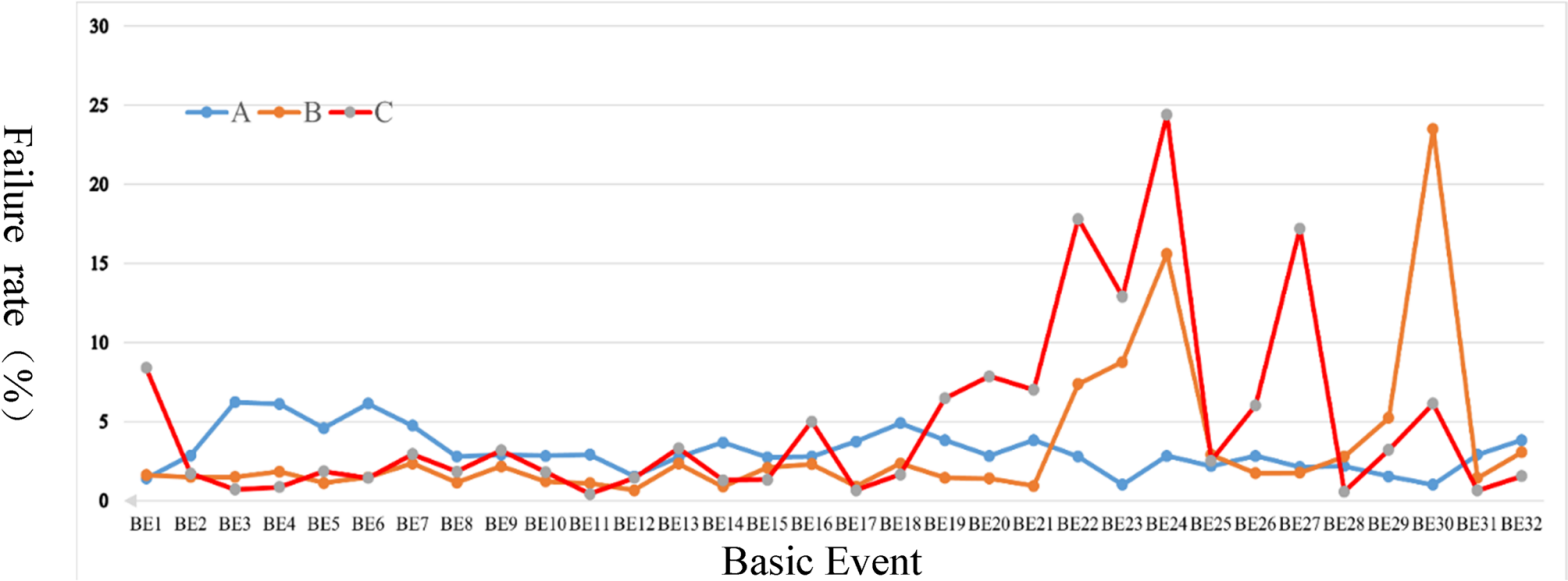
Posterior probability distribution comparison chart of basic events in failure of three groups of monitors.

To examine the main causes of monitor failures at each stage, the model assumes a 100% monitor failure rate, as shown in Fig. 6. Bayesian networks calculate the posterior probabilities of each basic event occurring when the top event happens. Figure 7 displays the basic event distributions causing monitor failures for groups A (1–5 years), B (6–10 years), and C (11–15 years).

Group A (1–5 years) exhibits an average failure rate of 3.22%, with a median of 2.83%. Group B (6–10 years) has an average failure rate of 3.29%, with a median of 1.77%. Group C (11–15 years) presents an average failure rate of 4.79%, with a median of 2.53%. The average failure rate generally rises with usage, though the median doesn’t entirely reflect this increase. There’s a slight average failure rate rise from 1 to 5 to 6–10 years (3.22–3.29%) and a notable increase from 6 to 10 to 11–15 years (3.29–4.79%).

Analysis of high-risk components indicates:

1. Group A (1–5 years): Highest failure rates in BE3, BE4, BE6, BE7, and BE18.

(2) Group B (6–10 years): Highest failure rates in BE22, BE23, BE24, BE29, and BE30.

(3) Group C (11–15 years): Highest failure rates in BE20, BE22, BE23, BE24, and BE27.

These high-risk components should receive more frequent inspections and maintenance. The correlation between groups A and B is low (0.11), suggesting different early-to-mid stage failure patterns. There’s a higher correlation between groups B and C (0.68), indicating some similarity in mid-to-late stage failure patterns, while the correlation between groups A and C is very low (−0.07), highlighting significant early-to-late stage differences. Analyzing failure rate changes shows that 17 components increased failure rates over time, and 15 decreased, with no components maintaining completely stable failure rates, indicating that most components’ failure rates change with usage, though the direction varies.

## Practice and application

After the theoretical research, a one-year practical study was conducted in 3201 hospital to verify its feasibility concerning maintenance costs. When a monitor malfunctions, the probabilities of different basic events guide the advance preparation of spare components, allowing for immediate maintenance and replacement. Maintenance costs encompass: (1) part procurement costs; (2) downtime loss costs; (3) expedited procurement costs; (4) inventory management costs. The average cost for parts is 200 RMB, with downtime costing 10 RMB per hour; repairs take approximately one hour per component. Temporary parts take about three days to arrive, with expedited procurement incurring costs about 20% higher than normal. The hospital operates around 457 monitors, with 179 in the 1–5 year stage, 190 in the 6–10 year stage, and 88 in the 11–15 year stage. Based on the posterior probability analysis, spare parts were organized, as shown in the accompanying Table 9.

**Table 9:** Cost comparison table for monitor maintenance: with FTA-BN vs. without FTA-BN.

	With FAHP -FTA-BN					Without FAHP -FTA-BN (No Spares)
	Basic Events	Stock	Used	Bias	120,000	115,400
The cost of part procurement	BE1	14	16	−2		
	BE2	10	9	1		
	BE3	15	16	−1		
	BE4	16	18	−2		
	BE5	12	18	−6		
	BE6	16	17	−1		
	BE7	16	15	1		
	BE8	9	11	−2		
	BE9	13	10	3		
	BE10	9	12	−3		
	BE11	8	10	−2		
	BE12	6	10	−4		
	BE13	13	14	−1		
	BE14	10	8	2		
	BE15	11	9	2		
	BE16	14	13	1		
	BE17	10	9	1		
	BE18	15	18	−3		
	BE19	16	20	−4		
	BE20	15	15	0		
	BE21	15	14	1		
	BE22	35	30	5		
	BE23	30	27	3		
	BE24	57	64	−7		
	BE25	12	15	−3		
	BE26	14	12	2		
	BE27	23	28	−5		
	BE28	10	15	−5		
	BE29	16	18	−2		
	BE30	52	60	−8		
	BE31	9	8	1		
	BE32	15	18	−3		
Downtime Loss Cost	51,850					421,210
Expedited Procurement Cost	2,560					23,080
Inventory Management Cost	16,080					0
Total Cost	190,490					559,690

The specific calculation steps are as follows:

Step 1: Calculate the stock of spare parts (SSP) based on the quantity of monitors in each group and the posterior probability (PP).

19 $$\:\begin{array}{c}SSP\left(BEi\right)=Group\left(A\right)*PP\left(Ai\right)+\:Group\left(B\right)*PP\left(Bi\right)+\:Group\left(C\right)*PP\left(Ci\right)\end{array}$$

For example:

20 $$\:\begin{array}{c}SSP\left(\text{B}\text{E}1\right)=179*1.41\%+190*1.62\%+88*8.41\%=13.16\end{array}$$

Then, the number of spare parts prepared in accordance with BE1 is 14. The number of spare parts corresponding to other events can all be obtained by this calculation.

Step 2: Calculate the cost.

(1)With FAHP -FTA-BN:

21 $$\:\begin{array}{c}The\:cost\:of\:part\:procurement=\left(536+64\right)*200=120,000\end{array}$$

In this equation, 536 represents the quantity of spare parts prepared, and 64 stands for the number of spare parts procured through expedited procurement.

22 $$\:\begin{array}{c}Downtime\:Loss\:Cost=577\:*\:10\:+\:64*3*24*10=\text{51,850}\end{array}$$

In this equation, 577 is the number of spare parts actually used, and 64 is the number of spare parts procured through expedited procurement.

23 $$\:\begin{array}{c}Expedited\:Procurement\:Cost=64*200*20\%=2560\end{array}$$

24 $$\:\begin{array}{c}Inventory\:Management\:Cost=536*200*15\%=\text{16,080}\end{array}$$

(2)Without FAHP -FTA-BN (No Spares):

25 $$\:\begin{array}{c}The\:cost\:of\:part\:procurement=577*200=115,400\end{array}$$

26 $$\:\begin{array}{c}Downtime\:Loss\:Cost=577\:*\:10\:+\:577*3*24*10=421,210\end{array}$$

27 $$\:\begin{array}{c}Expedited\:Procurement\:Cost=577*200*20\%=\text{23,080}\end{array}$$

28 $$\:\begin{array}{c}Inventory\:Management\:Cost=0\end{array}$$

It can be known from calculations that after employing the FAHP -FTA-BN method and preparing spare parts in advance, the total cost will be significantly reduced by 66%. While downtime losses are challenging to quantify precisely, the estimated hourly loss, derived from clinical practice, serves as a reference point. Prompt repairs are crucial, as delays following a monitor failure can disrupt clinical operations, contributing to increased downtime costs. In the scenario where spare parts are available, the loss cost caused by downtime constitutes 27.2% of the total cost. Conversely, when there are no spare parts, it accounts for 75.5% of the total cost. Moreover, with adequate preparation, the unnecessary expenses associated with expedited procurement can be minimized, further optimizing maintenance budgets. After practical application, using FAHP -FTA-BN to determine the failure rates of various monitor components and preparing spare parts in advance can significantly reduce maintenance costs.

## Discussion

In this study, the multi-parameter monitor was selected as the research object, and its entire life cycle was divided into three stages. In the absence of historical maintenance data, the FAHP -FTA-BN method was utilized to explore the failure probabilities of the monitor at different usage stages, clarify the differences in failures at each stage, and analyze the key failure causes that should be prioritized in each stage. Subsequently, the analysis results were applied to practical maintenance work. A comparison of maintenance costs revealed that the method proposed in this study effectively reduces maintenance costs, thereby demonstrating its practical applicability. The obtained results provide valuable insights and have been further confirmed through empirical verification. The details are as follows:

(1) **Precise Quantification of Failure Probabilities.** The application of the FAHP -FTA-BN method enables precise quantification of failure probabilities, providing crucial data for formulating maintenance plans tailored to the different usage stages of the monitor. Such detailed data is vital for developing targeted maintenance strategies.

(2) **Accurate Identification of High-Risk Components.** Through in-depth analysis, distinct high-risk components have been accurately identified within each usage age group. This finding underscores the necessity of formulating customized maintenance plans for these high-risk components, which may involve more frequent inspections and preventive replacements to mitigate the risk of potential failures.

(3) **Dynamic Changes in Failure Modes.** This study reveals significant differences in failure modes during the early, mid, and late stages of the monitor’s life cycle. These findings clearly indicate the need to develop adaptive maintenance strategies that can flexibly respond to such changes, thereby enhancing the effectiveness of maintenance efforts.

(4) **In-depth Analysis of Component Aging Characteristics.** Over time different components exhibit varying failure rates. Special attention should be directed towards those components that demonstrate gradually increasing failure rates. It may be necessary to modify their designs or utilize high-performance materials to ensure the reliability of both the components and the overall device.

(5) **Detailed Consideration of Cost-Benefit Analysis**. This study conducted a comprehensive cost-benefit analysis by considering the replacement costs of components alongside their impact on the overall performance of the device. The results indicate that an optimal replacement strategy can be developed to achieve a delicate balance between maintenance expenditures and equipment reliability.

The scheme proposed in this study demonstrates its superiority over existing maintenance practices in several respects. In the absence of historical data, the method effectively leverages expert experience. Unlike previous studies that primarily employed qualitative or quantitative analyses in isolation^39,40^, this approach integrates both aspects, thereby providing a more holistic understanding of the failure mechanism. This integration is particularly evident in the more accurate identification of high-risk components and the formulation of targeted maintenance strategies. Additionally, in contrast to studies that did not adequately consider the changes occurring at different stages of the equipment life cycle^41^, this research underscores the importance of developing adaptive maintenance strategies informed by the observed variations in failure modes throughout the life cycle. This enables a more effective response to the unique challenges presented at each stage, thereby optimizing the maintenance process.

In contrast to some existing studies that may have overlooked the cost-benefit analysis aspect^42^, this study conducts a comprehensive analysis of the replacement costs of components and their impact on device performance. This approach facilitates the design of an optimal replacement strategy that achieves an ideal balance between maintenance expenditures and equipment reliability, which is a crucial factor in ensuring the long-term viability of medical equipment maintenance.

The empirical results of this study, particularly the high degree of alignment between projected and actual spare parts requirements, further validate the effectiveness of the FAHP -FTA-BN method. Consistent with findings reported in related studies^8,43^, this research confirms the feasibility of utilizing this method to reduce maintenance costs through optimized inventory and procurement processes. However, it is important to note that the method employed in this study may introduce a degree of subjectivity, potentially affecting the accuracy of the results. For instance, when identifying high-risk components based on expert opinions, variability in outcomes may arise due to differences in the professional knowledge and practical experience of the participating experts. Therefore, careful consideration of this subjectivity is essential when interpreting the results of this study.

In the field of medical equipment maintenance, particularly concerning predictive maintenance, future research holds significant potential for further advancements. Building on the findings and limitations of this study, it is noteworthy that numerous researchers have proposed the concept of predictive maintenance^5,44^, which aims to forecast the future condition of medical equipment using data-driven methods to facilitate planned and targeted maintenance. By leveraging fault trees in conjunction with historical maintenance data, it is possible to expand the fault knowledge base and develop an expert system that integrates data-driven insights with experiential knowledge. This system seeks to enhance the accuracy and reliability of diagnostics through the optimal fusion of historical data and expert input. For instance, by combining machine learning algorithms with expert rules, the expert system may more accurately identify complex fault patterns and provide tailored maintenance recommendations. Furthermore, continuous improvement of the cost-benefit analysis in medical equipment maintenance is essential. Future research could focus on developing more sophisticated models that consider additional factors, such as the impact of equipment downtime on patient care, the costs associated with lost productivity due to equipment failures, and the long-term financial implications of various maintenance strategies. Incorporating these factors into the cost-benefit analysis will enable the design of more comprehensive and accurate maintenance strategies, ensuring the economic feasibility and effectiveness of medical equipment maintenance.

## Conclusion

Using the multi-parameter monitor as a case study, this research analyzed the fault conditions of the monitor across various trial stages by FAHP -FTA-BN, despite the absence of maintenance historical data. The following conclusions were drawn:

1. This method has been demonstrated to be both practical and feasible. It is particularly beneficial for medical institutions in developing countries and many small medical facilities, as it effectively manages the maintenance costs of medical equipment within the constraints of limited resources.

(2) This approach has facilitated both qualitative and quantitative analyses of medical equipment faults. It has accurately assessed fault probabilities at different usage stages, identified high-risk components, tracked changes in fault modes, understood the aging characteristics of components, formulated preventive maintenance strategies, and conducted cost-benefit analyses. Collectively, these elements provide essential technical support for maintenance modes and plans, thereby enhancing maintenance efficiency.

(3) Through a combination of practical experience and theoretical research, ensuring the availability of necessary spare parts in advance can significantly lower maintenance costs, thereby supporting the high-quality development of hospitals.

This study also opens new avenues for research into the maintenance of medical equipment in future medical institutions, including intelligent fault diagnosis, enhancement of the hybrid model, and improvement of cost-benefit analysis. These initiatives aim to further increase the effectiveness and economic viability of medical equipment maintenance, building on the foundation established by this study.

## Data Availability

The data presented in this study are available on request from the corresponding author. The data are not publicly available due to privacy and ethical.
